# Malaria vector research and control in Haiti: a systematic review

**DOI:** 10.1186/s12936-016-1436-x

**Published:** 2016-07-22

**Authors:** Joseph Frederick, Yvan Saint Jean, Jean Frantz Lemoine, Ellen M. Dotson, Kimberly E. Mace, Michelle Chang, Laurence Slutsker, Arnaud Le Menach, John C. Beier, Thomas P. Eisele, Bernard A. Okech, Valery Madsen Beau de Rochars, Keith H. Carter, Joseph Keating, Daniel E. Impoinvil

**Affiliations:** Programme National de Contrôle de la Malaria, Port-au-Prince, Haiti; Division of Parasitic Diseases and Malaria, Center for Global Health, Centers for Disease Control and Prevention, Atlanta, GA USA; Clinton Health Access Initiative, Boston, MA USA; Division of Environment & Public Health, Department of Public Health Sciences, University of Miami Miller School of Medicine, Miami, FL USA; Center for Applied Malaria Research and Evaluation, Department of Tropical Medicine, Tulane School of Public Health and Tropical Medicine, New Orleans, LA USA; Department of Environmental and Global Health College of Public Health and Health Professions, Emerging Pathogens Institute, Gainesville, FL USA; Department of Health Service Research Management and Policy of College of Public Health and Health Professions, Emerging Pathogens Institute, Gainesville, FL USA; The Carter Center, Atlanta, GA USA; Department of Communicable Diseases and Health Analysis, Pan American Health Organization/World Health Organization, Washington, DC USA

**Keywords:** Haiti, Malaria, Anopheles, Vector control

## Abstract

**Background:**

Haiti has a set a target of eliminating malaria by 2020. However, information on malaria vector research in Haiti is not well known. This paper presents results from a systematic review of the literature on malaria vector research, bionomics and control in Haiti.

**Methods:**

A systematic search of literature published in French, Spanish and English languages was conducted in 2015 using Pubmed (MEDLINE), Google Scholar, EMBASE, JSTOR WHOLIS and Web of Science databases as well other grey literature sources such as USAID, and PAHO. The following search terms were used: malaria, Haiti, *Anopheles*, and vector control.

**Results:**

A total of 132 references were identified with 40 high quality references deemed relevant and included in this review. Six references dealt with mosquito distribution, seven with larval mosquito ecology, 16 with adult mosquito ecology, three with entomological indicators of malaria transmission, eight with insecticide resistance, one with sero-epidemiology and 16 with vector control. In the last 15 years (2000–2015), there have only been four published papers and three-scientific meeting abstracts on entomology for malaria in Haiti. Overall, the general literature on malaria vector research in Haiti is limited and dated.

**Discussion:**

Entomological information generated from past studies in Haiti will contribute to the development of strategies to achieve malaria elimination on Hispaniola. However it is of paramount importance that malaria vector research in Haiti is updated to inform decision-making for vector control strategies in support of malaria elimination.

## Background

The island of Hispaniola remains the last vestige of endemic malaria transmission in the Caribbean [1, 2]. Both Haiti and the Dominican Republic (DR) have recently committed to achieve transmission interruption with the goal of no new malaria cases by 2020 [2, 3]. The World Health Organization (WHO) malaria elimination certification process will require an “absence of clusters of three or more epidemiologically-linked autochthonous malaria cases due to local mosquito-borne transmission, nationwide for three consecutive years” [4, 5]. While the vision for malaria elimination is shared in both Haiti and the DR, Haiti experiences a greater burden of both transmission and disease [2].

Malaria transmission in Haiti is low in relative and absolute terms [6–8]. In 2011, a national survey reported a prevalence of malaria parasitaemia in all ages of less than 1 % [2, 9, 10]. Currently, microscopy observations and polymerase chain reaction (PCR) amplification studies show that *Plasmodium falciparum* is the dominant parasite species transmitted in Haiti [9, 11]. *Plasmodium vivax* is believed to be mostly imported rather than locally transmitted [2] while *Plasmodium malariae* transmission persists but is low [11].

Forty percent of the landscape in Haiti is at elevations greater than 458 m above sea level (a.s.l.). The terrain in Haiti is mainly rough and mountainous, with numerous springs and seepage areas throughout the low-lying areas. Much of the low-lying area is also farmed and contains irrigation canals, further creating conditions suitable for mosquito proliferation. It has also been noted that salt flats sometimes contain tidal zone springs and lines of seepage that encourage mangrove swamp formation; this brackish water environment is an ideal mosquito habitat, especially during the rainy season or when foot paths are created [12].

Haiti has a tropical semiarid climate. Twenty-year (1990–2009) climate data for Haiti show estimated average temperatures range from 23 °C in January to 27 °C in August; precipitation in Haiti is bimodal, with a rainfall peak in May (~242 mm) and in November (~280 mm) [13]. Peak malaria transmission corresponds to the rains on the island, with regional and temporal variation as a function of onset, duration and abundance of rain. Natural hazards such as hurricanes and earthquakes have also been associated with increased malaria transmission, or concern for increased transmission [2, 14, 15]. Because Haiti and DR share borders, movement between the two countries occurs regularly. Mostly this consist of Haitian migrants looking for work in the DR [2]. This movement may also contribute to the geographic scope of malaria on the island.

Prior to the malaria eradication campaigns of the 1950s/60s, sanitarians in Haiti used larval control such as drainage, filling and oiling as the main malaria intervention [16]. In 1958, the Government of Haiti (GOH) declared malaria to be an urgent problem of national interest and created the Service National d’Eradication de la Malaria (SNEM); in 1961, a co-operative agreement was established between the GOH, the World Health Organization (WHO), United States Agency for International Development (USAID), and the United Nations Children’s Fund (UNICEF) to provide financial and technical assistance for malaria elimination [16, 17]. When the malaria eradication campaign was fully implemented in Haiti in 1961, the Haitian malaria strategy largely abandoned larval control and adopted the strategies of indoor residual spraying (IRS) with DDT and mass drug administration (MDA), as it was thought globally that these tools were enough to achieve eradication [18]. Malaria was almost eliminated from Hispaniola. In Haiti, the slide positivity rate was reduced from 4.0 % in 1964 to 0.2 % in 1968 [19]; in the DR, reported cases reduced from greater than 5000 cases in 1960 to 21 cases in 1968 [2]. However, both Haiti and the DR could not sustain the gains. In 1970, USAID decreased financial support to Haiti, which was deemed insufficient to eliminate malaria; hence, emphasis was placed upon trying to maintain gains rather than eliminate malaria altogether [2]. SNEM formally changed its focus from eradication to control in 1979, additionally changing their name to the Service National des Endémies Majeures (SNEM) [17]. In 1988, SNEM was officially dismantled and malaria control in Haiti was restricted to response to epidemics and natural disasters [2]. In 2005, the Programme National de Contrôle de la Malaria (PNCM)/National Malaria Control Programme (NMCP) was officially created allowing the re-launch of routine anti-malaria activities with support from donor agencies [3].

At present there is renewed binational interest in malaria elimination on the island of Hispaniola. Because information on malaria vector distribution, bionomics and control in Haiti is limited, the current literature on malaria vector dynamics was systematically reviewed, with the goal of informing malaria elimination strategy development. The purpose of this paper is to: (1) chronicle malaria vector-related research and programme monitoring in Haiti; (2) identify gaps in malaria vector control research, and; (3) discuss vector control approaches that may help interrupt transmission in Haiti given current mosquito dynamics and available vector control tools.

## Methods

A systematic electronic search of literature published between January 1940 and September 2015 in the English, French and Spanish languages was conducted using Pubmed (MEDLINE), Google Scholar, EMBASE, JSTOR, WHOLIS and Web of Science databases. The following search terms (or their French and Spanish equivalents) were used: malaria, Haiti, Hispaniola, *Anopheles*, and vector control. All results were initially reviewed for mosquito bionomics (larval ecology adult ecology) and vector control relevance based on the title and abstract. Relevant publications were further reviewed using the full text to determine whether primary or secondary data on the distribution, bionomics, epidemiology, insecticide resistance, vector competence, sero-epidemiology or control of malaria vectors existed. These terms were selected for their epidemiological relevance to malaria elimination in Haiti. The term, genetics, was not included in the review search. The grey literature and programmatic documents were also searched using Google, Google Scholar and other Open Content search engines using the same search terms.

## Results

Table 1 illustrates the results of the search. A total of 132 papers were identified as potentially relevant; a total of 40 papers and reports met the criteria. The other 92 documents did not explicitly contain data or information related to mosquito bionomics or control. Much of the literature on mosquito behaviour and control has been intermittent in Haiti with large time gaps between publications as shown in Table 1.

**Table 1:** Summary of references

Category	Sub-category	References			
		Prior to eradication (<1962)	Eradication years (1962–1970)	Post-eradication (1971–2000)	Recent (2000–present)
Mosquito distribution (6)	–	[12]	–	[16, 20, 21, 25, 39]	–
Larval ecology (7)	–	–	–	[25, 30, 31]	[35–37, 41, 42]
Adult ecology (16)	Resting (3)	–	[46]	[16, 30]	–
	Host-seeking/biting (5)		[27]	[25, 30, 31, 47]	
	Parity/gonotrophic cycle/longevity (3)	–	–	[25, 30, 31]	–
	Host selection (2)	–	[52]	[30]	–
	Vector competence (3)	–	–	[32–34]	–
Ento-epidemiology (3)	–			[25, 28, 29]	
Insecticides/resistance (8)	–	[62]	[46, 63–65]	[16, 67, 68]	
Sero-epidemiology (1)					[61]
Vector control (16)	Larval source management (LSM) (3)	–	[72, 73]	[19]	–
	Indoor residual spraying (IRS) (5)	–	[46, 63, 65, 75]	[16]	
	Space spraying (7)	–	–	[19, 53, 66, 68, 78–80]	–
	Bed nets (1)	–	–		[69]

Documents in French [25, 26, 31, 39], documents in Spanish [42, 52]

Numbers in parentheses are total number of references for each category; numbers in brackets correspond to the reference citation

### Malaria vector distribution in Haiti

Six species of anophelines have been reported to occur on Hispaniola: *Anopheles albimanus*, *Anopheles argyritarsis*, *Anopheles crucians*, *Anopheles grabhamii*, *Anopheles pseudopunctipennis* and *Anopheles vestitipennis* [20, 21]. Of the six species, *A. albimanus* is thought to be the principal vector of malaria in Haiti [12, 16, 22] and, based on the review of the known literature, is the only species reported to have fulfilled all the criteria of vector incrimination in this country [23, 24], specifically: (1) association in time and space between the vector and human malaria cases [12, 25–30], (2) evidence of direct contact between the *Anopheles* species and humans [27, 28, 31], (3) evidence that the *Anopheles* species harbours malaria sporozoites in the salivary glands under natural conditions [25, 26, 29], and (4) demonstration of transmission of the pathogen by the vector under experimental conditions [32–34].

*Anopheles crucians, A. grabhamii, A. pseudopunctipennis* and *A. vestitipennis* have been identified infrequently relative to *A.albimanus*. Studies over the last 10 years report no *Anopheles* other than *A. albimanus* [35–37]. There are doubts that *A. argyritarsis* actually occurs on Hispaniola. It has been suggested that any identification *A. argyritarsis* may be *A. albimanus* whose hind tarsi have broken off [38]. More recent publications do not list *A. argyritarsis* as a species found in Haiti [12, 16, 25].

**Fig. 1:**
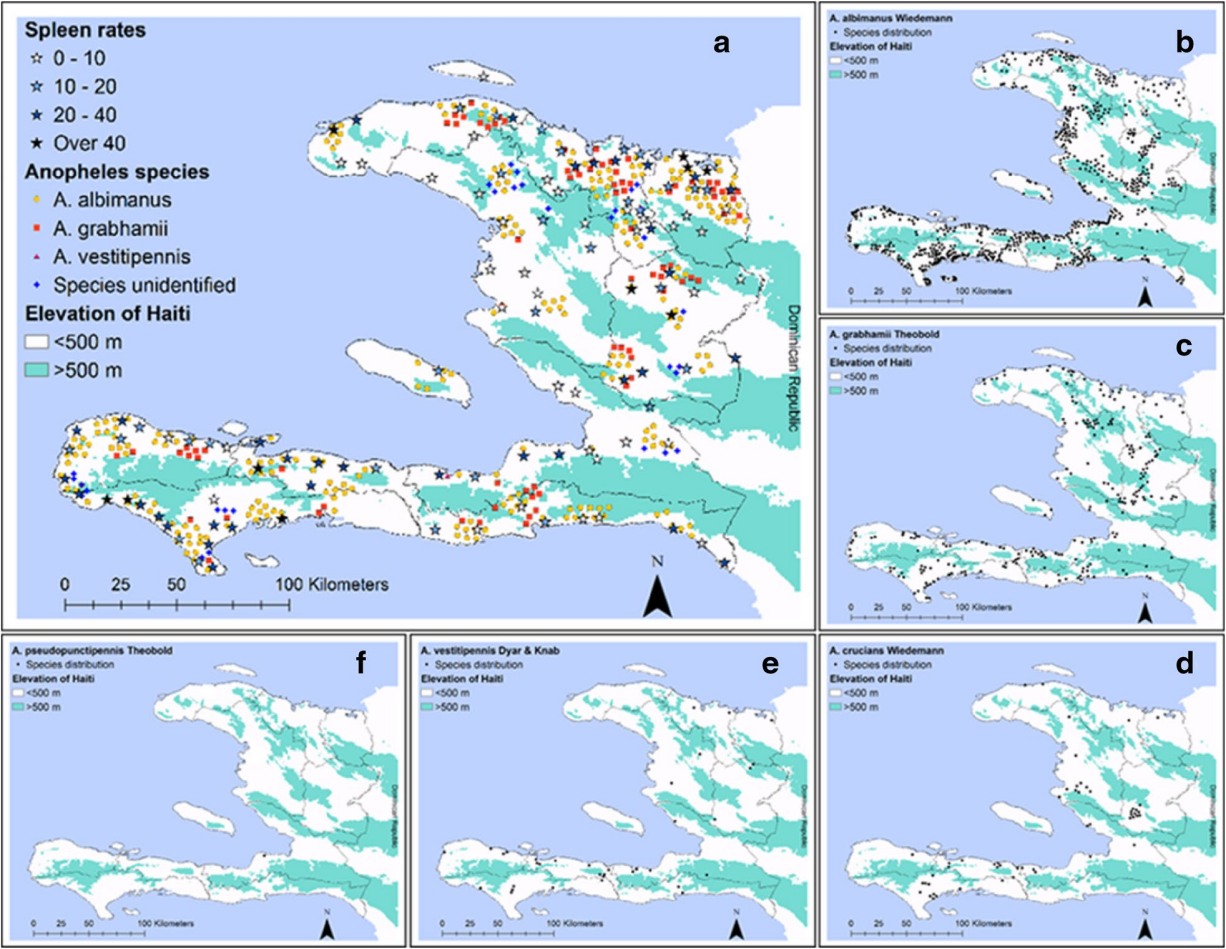
Maps of Haiti with malaria and entomological data. Points have been georeferenced and digitized from publication maps using current departmental base map of Haiti—*points* represent localities. **a** Distribution of school spleen enlargement rates and *Anopheles* larval sites in the Republic of Haiti. **b**–**f** Distribution of *Anopheles albimanus*, *A. grabhamii*, *A. crucians, A. vestitipennis*, and *A. pseudopunctipennis*, respectively. **a** Reproduced with permission from rights holder, the American Society of Tropical Medicine and Hygiene [12]. **b**–**f** Reproduced from SNEM entomology records—1979–1984 [16]

Paul and Bellerive [12, 16] published the first national distribution map of malaria cases and malaria vectors in Haiti; this study was conducted across Haiti by the Service National d’Hygiène of Haiti and The Rockefeller Foundation from 1940 to 1942. The survey was limited to an examination of school children to determine the rate of splenomegaly and associated parasite positivity by blood smears, as it was thought this would give an adequate representation of malaria prevalence. The malaria parasite prevalence for all species was 31 %. Of 5507 positive parasite smears examined, 86.6 % were *P. falciparum*, 8.9 % were *P. malariae*, 1.9 % were *P. vivax* and 2.6 % were mixed infections. Mosquito surveys primarily consisted of a brief reconnaissance for anopheline larval sites around the surveyed schools during the long dry season. Limited adult collections in houses were largely unsuccessful. *Anopheles albimanus* was the mosquito most commonly found and identified followed by *A. grabhamii*. *Anopheles vestitipennis* was found only in Petit-Goâve during the survey. Figure 1a shows the distribution of spleen enlargement rates and *Anopheles* species. In 1986, *A. pseudopunctipennis* was discovered in Bellevue, Haiti, a coastal area south of Port-au-Prince in the Ouest Department [39]. A report by Feinstein in 1995, based on SNEM records, provided additional maps of malaria vector distribution expanding the number of species and locations of sightings between 1960 and 1988 (Fig. 1b–f) [16]. The SNEM records generally reported *Anopheles* occurring below 500 m a.s.l. However, these records indicated that *A. grabhamii* and *A. albimanus* were collected at elevations up to 725 and 762 m a.s.l., respectively [16].

### Larval mosquito ecology

Specific habitats of anophelines in Haiti are variable, but general characteristics likely include fresh or semi-brackish water, no shade to fully shaded habitats, and little to no pollution. Examples of habitats include rice-fields and ponds [30]. Larval habitats appear to be shared among four of the five species. Although *A. pseudopunctipennis* has not been identified in larval habitats, only collected as adults [39], it is likely larvae of these species would also be found in habitats similar to the other anopheline mosquito species in Haiti [40]. Further, a recent study characterizing aquatic mosquito habitat, natural enemies, and immature mosquitoes in the Artibonite Valley, Haiti found *A. albimanus* larvae occurring in temporary, semi-permanent and permanent groundwater habitats including hoof/footprints, ditches, rice fields, and ground pools [41]. Co-occurrence was a notable feature of the habitats, with 42.9 % of *Anopheles*-positive sites also having *Culex* species.

In 2011, as part of post-earthquake assistance from Cuba to Haiti, the Cuban medical brigade reported the distribution and biological aspects of the most well-known mosquito vectors of Haiti [35, 36, 42]; this report showed a wide distribution of *A. albimanus* across Haiti. In 2013, a study looking at urbanization, land-use and larval abundance of mosquitoes in the city of Cap-Haïtien and the coastal village of Caracol, collected *A. albimanus* from a highly brackish riverbed found in an agricultural area that housed banana plants [37]. *Anopheles albimanus* from this collection accounted for <1 % (8 of 876) of the total number of larval mosquitoes collected, suggesting low abundance of *A. albimanus* in urban areas.

In 1988, a study on field-collected *A. albimanus* from Bellevue, Haiti reared on site under ambient conditions found that the duration of mosquito immature development (eggs-to-larvae-to-pupae-to-adults) was approximately 15-days with average daytime temperatures of 27 °C and night-time temperatures of 21 °C [25, 31]. In the same study, the findings were compared to mosquitoes reared in an insectary in Port-au-Prince, which showed a larval development time of approximately nine days with average daytime temperature of 30 °C and night-time temperature of 27 °C.

### Adult mosquito ecology

#### Resting

Studies on the resting behaviour of malaria vectors in Hispaniola are limited. Knowledge of mosquito resting behaviour is essential for implementing vector control interventions that use spray application of insecticides; control of mosquitoes that are endophilic (i.e. tendency to rest indoors) for example could be targeted using indoor residual spraying, while control of mosquitoes that are exophilic (i.e. tendency to rest outdoors) could be targeted using outdoor space spray approaches or larval control.

Diurnal resting habits of mosquitoes refers to sites (usually outdoors) selected by mosquitoes when they are not actively seeking hosts, sugar, or oviposition habitat [43]; this is usually the period between dawn and dusk for nocturnal mosquitoes. A study in DR from 1987 to 1988 did not find any adult anophelines in potential diurnal resting sites consisting of vegetation, river banks, tree trunks, piles of hollow blocks or corrals [30]. A PAHO report from Haiti summarizing a decade of malaria control from 1978 to 1988 stated that the SNEM entomological teams found anophelines resting in ground holes, grass and other sites [16].

Indoor resting habit of mosquitoes refers to the time when a vector enters a home, bites a host and rests in that home for some duration [43]. In 1987–1988, studies on indoor resting in Dajabón, a town bordering northeastern, Haiti found that within houses known to have harboured mosquitoes in the past, only two *A. albimanus* and two *A.vestitipennis* mosquitoes were found in 25 houses using mouth aspirators; only six *A. albimanus* were found in 35 houses using pyrethrum spray catches (PSCs), and only 17 *A. albimanus*, 17 *A. vestitipennis* and one *A. crucians* were captured over nine trapping nights using window exit traps [30], suggesting limited resting indoors. Despite its trend towards exophily, it is likely that immediately after an indoor bite a fully engorged mosquito will rest, even briefly, on insecticide-treated wall before egressing outwards; studies in countries other than Haiti, have found *A. albimanus* to rest 8–14 min on treated walls [43–45]. However, a study in four villages near the port city of Cap Haitien, Haiti (Morne Anglais, Belle Hotesse, La Fond and St. Michel) compared the resting and biting rates of *A. albimanus* [28]. When the product of the rest-to-bite ratio (0.39) was calculated (i.e. number of freshly blood fed mosquitoes resting on walls divided by the number biting mosquitoes) and the indoor-to-outdoor bite ratio (0.31), derived from the study, a value of only 12 % was found, suggesting that in these areas a small percentage of the biting *A. albimanus* mosquito population would actually be in direct contact with insecticides on the walls after residual spraying.

Taylor et al. [43, 46] also noted that more than 50 % of *A. albimanus* rested at heights of less than three feet in experimental hut studies with no differences in heights before and after insecticide application to the walls. This result suggests that high wall areas in the home would not require spraying, which could lower the indoor residual spraying cost.

#### Host-seeking/biting

**Fig. 2:**
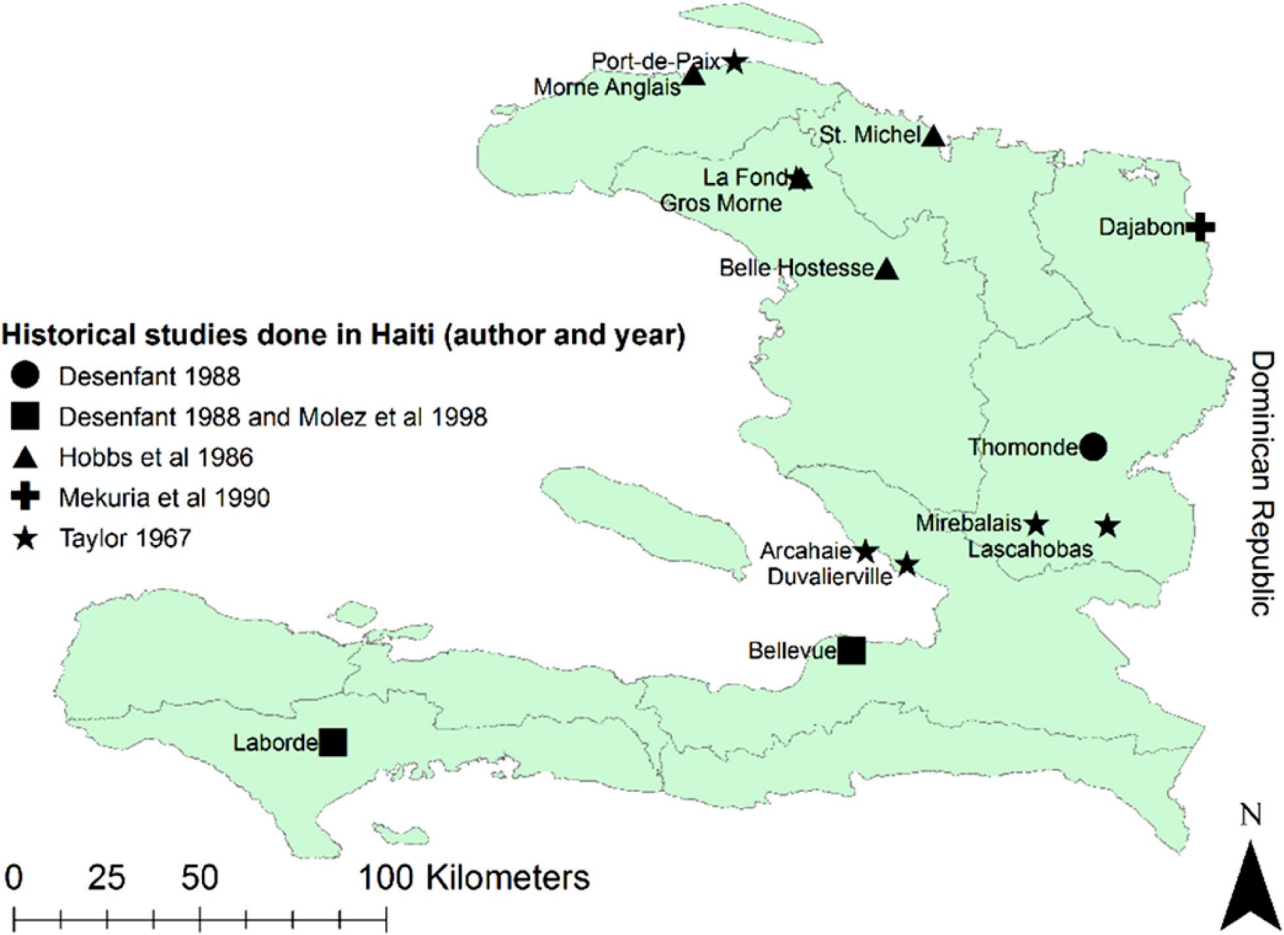
Map showing sites where known mosquito behaviour studies have occurred historically

Mosquito biting behaviour includes biting seasonality, diel biting activity (i.e. peak biting in a 24-h cycle) and preferred biting location relative to a house (indoor vs outdoor). Such studies have been conducted in several areas of Haiti and during different time periods (Fig. 2). Still, these data are somewhat dated and limited with the most recent of such studies being conducted from 1986 to 1988 [25, 28–31].

One of the first published studies on seasonal malaria vector biting behaviour was by Taylor in 1966 [27]. This study, conducted from 1963 to 1964, showed variable seasonal biting behaviour of *A. albimanus* at six different sites in the communes of Mirebalais, Lascahobas, Duvalierville, Port-de-Paix, Gros Morne and Arcahaie. Human baited collections were done near temporary, semi-permanent, and permanent water bodies. The association between seasonal biting and rainfall was highly variable, with the exception of Port-de-Paix. It was suggested that the lack of association between biting and rainfall may be due to rice cultivation practices in permanent larval development sites, in which the mosquito population would be less reliant on rainfall. In contrast, studies by Desenfant [25] and Molez et al. [31], conducted in 1986–1987, found that June and October through December were the periods where peak biting occurred. Based on their findings, Desenfant [25] consolidated peak mosquito biting to the following months: October–December high biting (wet season); January–May low biting (dry season); June high biting (wet season); July–September low biting (dry season).

Studies on peak biting times have mostly been done for *A. albimanus*, and *A. vestitipennis* [30]. Reported peak biting times for *A. albimanus* were variable. Studies by Taylor in 1963–1964 [27] and Hobbs et al. in 1983–1984 [28] observed a biting cycle with peak biting time occurring early in the evening (19:00–23:00) and biting activity steadily declining thereafter. In the Mekuria et al. study in 1987–1988 [30], results similar to those of Taylor [27] and Hobbs [28] were observed, with an additional second small peak from 04:00 to 05:00 followed by a sharp decline. In the Desenfant [25] and Molez et al. [31] study from 1986 to 1987, bell-shaped curve biting cycles were observed with the majority of biting activity occurring generally from 21:00 to 02:00 [25, 31]; these studies took place in the south of Haiti, while studies by Hobbs et al. [28] and Mekuria et al. [30] took place in the north of Haiti, suggesting geographical variation in behaviour. The six sites Taylor investigated had wide latitudinal distribution; biting cycles for individual sites were not reported. Differences between these studies may be due to variation in night-time behaviour of hosts causing increased or decreased availability to mosquitoes, changes in mosquito behaviour driven by insecticide exposure or other factors such as microclimate variation impacting the diel biting activity.

There is a general trend for *A. albimanus* to bite more outdoors than indoors in Haiti (Table 2); however, the biting location of *A. albimanus* seems to be variable with areas, such as Dajabón in the DR reporting 30-fold higher biting outdoors than indoors and some areas, such as Laborde in Haiti showing only 10 % higher biting outdoors than indoors.

**Table 2:** Outdoor-to-indoor biting ratios of *Anopheles albimanus* derived from human-landing catch and light trap studies in Haiti

Author and year	Reference	Location	Outdoor-to-indoor biting ratio^f^
Taylor 1976	[27]	Various sites in Haiti^a^	3.87
Hobbs et al. 1986	[28]	Various sites in Haiti^b^	2.10
Mekuria et al. 1990^c^	[30]		
*An. albimanus*		Dajabón, DR	30.22
*An. vestitipennis*		Dajabón, DR	16.19
Desenfant 1988/Molez et al. 1998	[25, 31]		
–		Bellevue, Haiti—EM48	1.16
–		Bellevue, Haiti—EM61	1.95
–		Laborde, Haiti	1.10
Sexton et al. 1986^d^	[47]		
HLC		Various sites in Haiti^b^	5.60
UV light trap		Various sites in Haiti^b^	3.88
CDC light trap		Various sites in Haiti^b^	0.29

^a^Sites included: the communes of Mirebalais, Las Cahobas, Duvalierville, Port-de-Paix, Gros Morne and Arcahaie

^b^Sites included: Four villages of Morne Anglais, Belle Hotesse, La Fond and St. Michel

^c^This study provided results for *An. albimanus* and *An.* *vestitipennis*

^d^This study provided results for HLC, UV light traps and CDC light traps

^e^This study provided results for UV light traps

^f^The ratios were calculated by adding one to each outdoor and indoor biting value calculating the outdoor-indoor ratio then averaging the ratio

Light traps are often used to sample mosquitoes as a replacement for traditional human landing catches to circumvent problems in the field such as collector fatigue, human attractiveness differences and exposure to potentially infected mosquitoes. A study by Sexton et al. [47] conducted in northern Haiti from 1983 to 1984 compared *A. albimanus* catches from human landing catches (HLC), the Updraft ultraviolet (UV) light traps (UVLT)—a new trap at the time which uses UV light, and the miniature CDC light traps (CLT)—a standard light trap which uses incandescent light. The study found the UVLT caught more *A. albimanus* than HLC, and HLC subsequently caught more *A. albimanus* than CLT, both outdoors and indoors. However, CLTs still caught more *A. albimanus* indoors than outdoors (Table 2). The authors suggested this may have been due to contrast between the light emitted by the different traps (UV vs incandescent) and background illumination. The author also suggested updraft or downdraft movement of the air current from the trap may also account for this difference. Nonetheless, results from UV light trap reinforce the premise of more biting outdoors than indoors.

Behavioural studies are currently ongoing in Dame Marie, Grand’Anse Department, a town at the tip of the southern peninsula of Haiti and Ouanaminthe, Nord-est Department, Haitian border town with DR; preliminary results in Ouanaminthe show similar result as Taylor, Hobbs et al. and Mekeuria et al. where biting occurs early and declines through the evening, while preliminary result of in Dame Marie shows peak biting between 10:00 p.m.–12:00 a.m.; both studies show higher biting outdoors than indoors (unpublished data).

#### Parity/gonotrophic cycle/longevity

Parity studies are useful for understanding transmission dynamics, particularly in reference to vector control because older female mosquitoes that have laid eggs (parous) are more likely to transmit malaria parasites than younger ones which have not laid eggs (nulliparous) [48]. Parity rates are calculated as the number of mosquitoes that are parous divided by the total number of mosquitoes dissected. Studies by Mekuria et al. [30] found the overall parity rates from *A. albimanus* samples collected over a year from outdoor human baits and light traps to be 37.3 % (n = 566). For *A. vestitipennis*, the parity rates was 20.7 % (n = 169) [30]. Indoor parity rate for *A. albimanus* and *A. vestitipennis* was 33.3 % (n = 21) and 35.7 % (n = 56), respectively [30]. When mosquito biting data from the human bait catch-nights was divided into four quarters of the night (Q_1_: 18:00–21:00; Q_2_: 21:00–00:00; Q_3_: 00:00–03:00; Q_4_: 03:00–06:00) significant differences in the parity distribution (Q_1_% parous = 39, n_1_ = 90; Q_2_% parous = 45, n_2_ = 230; Q_3_% parous = 30, n_3_ = 135; Q_4_% parous = 33, n_4_ = 84) were observed. Parous females comprised a higher proportion of the samples captured in the first two quarters compared to last two quarters. The authors do not provide an explanation for this observation, but this difference in biting may be attributed to the diel emergence (peak activity in a 24-period cycle) of adult mosquitoes from larval sites or the interaction of other behaviours with parity status and blood-feeding such as sugar-feeding or mating behaviour. Desenfant [25] provides the most comprehensive study on mosquito parity in Haiti showing seasonal biting, external/internal biting behaviour and parity for three different sites. Results of his analysis were heterogeneous by site varying in biting within and between nulliparous/parous mosquitoes and external/internal biting location.

The duration of the gonotrophic cycle, defined as the time between mosquitoes taking a blood meal and laying a batch of eggs, is modelled in malaria transmission because it suggests how many blood meals will be taken from a host until it is ready to transmit. The gonotrophic cycle for *A. albimanus* in Dajabón was 2.6 days [30], while the gonotrophic cycle of *A. albimanus* from Bellevue was 5.16 days [25]. Assuming a malaria parasite incubation in mosquitoes of 9–12 day; mosquitoes acquire malaria parasites on their first feed; and gonotrophic concordance (i.e. mosquitoes take only one blood meal at set point of each egg development cycles), this would suggest that in Dajabón mosquitoes would take three to four blood meals before transmitting. In Bellevue, mosquitoes would take two to three blood meals before transmitting. However it is thought that *A. albimanus* violate these assumption of gonotrophic concordance by taking multiple meals during a gonotrophic cycle [49, 50] or exhibit two gonotrophic cycles; shorter for parous mosquitoes and longer for nulliparous mosquitoes [51]. The study in Bellevue suggested that nulliparous *A. albimanus* required a second blood-meal to achieve fully reproductive capacity [25], hence the difference in gonotrophic cycles between Bellevue and Dajabón. When using the gonotrophic cycle and parity rates to calculate daily survival rate, Desenfant [25] reported a survival rate of 0.88 while Mekuria et al. [29] reported a survival rate of 0.68.

#### Host selection

**Fig. 3:**
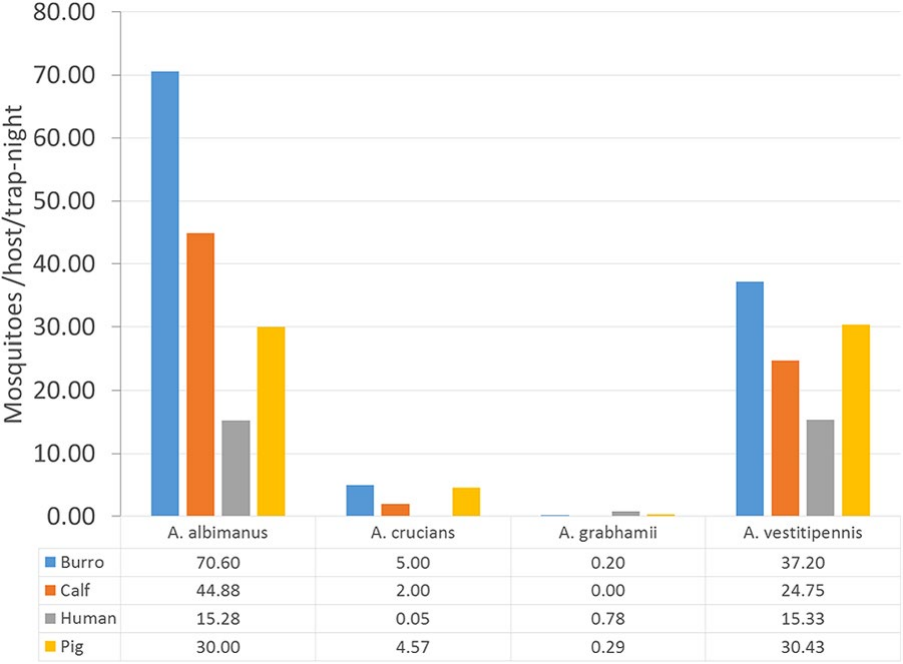
*Anopheles* species and abundance from animal-baited and human-baited traps in Dajabón, Dominican Republic in 1988. Number in table below are mosquito density per trap-nights for the burro, calf, human and pig are 5, 8, 10 and 7 nights, respectively. Reproduced with permission from rights holder, American Mosquito Control Association [30]

Studies by Ricciardi in 1971 [52] and Mekuria et al. in 1990 [30] are the only known published data on *Anopheles* host selection on Hispaniola. The study by Ricciardi used precipitin test and suggested that in areas where there is intensive livestock production, the degree of anthropophily of *A. albimanus* can be very low (9 meals on humans for 205 meals analysed) [52]. In the study by Mekuria et al., using an animal-baited net trap, the number of *A. albimanus, A. crucians, A. grabhamii* and *A. vestitipennis* attracted to a burro, calf, and pigs was determined. In Fig. 3 the animal data was combined with human data from Mekuria et al. to see the trend in biting preference. For *A. albimanus* and *A. vestitipennis*, more mosquitoes were captured on burros than humans, calves or pigs. Very few *A. crucians* and *A. grabhamii* were captured, but *A. grabhamii* seemed to favour humans while *A. crucians* favoured burros and pigs (Fig. 3).

#### Vector competence

Vector competence for malaria is defined as the ability of a species to transmit malaria parasites and is determined either through infection experiments or observation of sporozoites in salivary glands of field-caught mosquitoes. Experimental infections of native mosquitoes from Haiti have only been done with *A. albimanus* [32–34]. These mosquitoes originated from Arcahaie, in the Ouest Department. Infection experiments generally showed low (0–20 %) infection rates with *P. falciparum* and *P. vivax* [32–34].

*Anopheles albimanus* sporozoite rates of 0.00 [28], 0.03 [29], 0.04 [53], 0.21 [25], and 2.02 [26] percent have been reported. Using sporozoite ELISA, *P. falciparum* sporozoite antigen was found in the study by Mekuria et al. [29] and *P. vivax* sporozoite antigen was found in the study by Perich et al. [53]. In the other studies detecting parasites in the mosquitoes, salivary gland dissection were done; the *Plasmodium* species was not identified [25, 26].

### Ento-epidemiology

Vectorial capacity (VC) and the entomological inoculation rate (EIR) are the principal measures for entomologically evaluating transmission epidemiology and estimating the impact of vector control interventions on transmission dynamics. VC is defined as the “average number of inoculations with a specified parasite, originating from one case of malaria in unit time that the population would distribute to man if all the vector females biting the case became infected” [54]. VC is derived by measurements of human-mosquito contact rate, daily mosquito survival rate and the extrinsic incubation period of the pathogen. EIR is a measure of malaria transmission intensity and is defined as the number of infectious bites per human per unit time (usually years).

On Hispaniola, studies by Desenfant [25], Hobbs et al. [28], and Mekuria et al. [29] provide estimates of VC and EIR (Table 3). In general, new world mosquitoes such as *A. albimanus* are often considered a less efficient vectors compared to vectors with higher anthropophilic, endophagic, and endophillic behaviour such as *Anopheles gambiae* [55]. However, the factors mostly contributing to this higher efficiency are greater anthropophily and increased survival in *A. gambiae* (Table 3) [43, 56, 57].

**Table 3:** Vectorial capacity (VC) and entomological inoculation rate (EIR) of *Anopheles albimanus* in Haiti and a neighbouring site in Dominican Republic

Location	Year of study	Biting rate (ma)^a^	Survival rate (p)^b^	Biting habit (a)^c^	VC^d^ (Inoculations^e^)	Sporozoite rate (%)^f^	EIR^g^
Rates in Haiti for *An. albimanus*							
Sites in northern departments (refer to Hobbs et al. 1986) [28]	1983–1984	2.51	NC	NC	NC	0.00	0.00
Dajabón, Dominican Republic [29]	1986–1987	15.7	0.68	0.031	0.02 (~2)	0.03	1.72
Bellevue, Haiti [25]	1986–1987	42.33	0.88	0.175	9.61 (~768)	0.21	32.45
Laborde, Haiti [26]	1986–1987	29.20	NC	NC	NC	2.02	215.29
Comparison: rates in Nigeria for *An. gambiae*							
Northern Kankiya, Nigeria [59]	1967	9.10	0.94	0.250	16.20 (~1300)	5.90	195.97

*NC* not calculated

^a^Bites/person/night

^b^Probability of daily mosquito survival

^c^Proportion of blood meals taken from humans to the total number of blood meals taken from any animal

^d^Daily inoculations per single malaria case

^e^Estimated *P. falciparum* reproduction rate: Total number of inoculations from a single malaria case (The basic reproduction rate values are derived on the assumption that a non-immune, untreated case of *P. falciparum* is infective to the vector for a total of 80 days)

^f^Number of mosquitoes positive of sporozoites per mosquito tested multiplied by 100

^g^Infectious bites per person per year

Vector competence is also thought to be significantly higher for *A. gambiae* compared to *A. albimanus.* For, example a study for *A. gambiae* showed an average infection rates as high as 62 % (range: 8–100 %) [58] compared to 0–20 % in *A. albimanus* [32–34]. From a 1993 review of *A. albimanus* bionomics, Haiti is one of two countries that had reported sporozoite rates of >2 % in *A. albimanus* in the Americas. Colombia is the other country that has reported a sporozoite rate in *A. albimanus* greater than two percent (2.7 %). El Salvador reported a sporozoite rate in *A. albimanus* of 1.59 %. Other studies in Belize, Costa Rica, Dominican Republic, Honduras, Jamaica, Mexico, Panama, and Venezuela show a sporozoite rate less than 1 % [43]. In contrast, reported sporozoite rates of ~6 % have been shown for *A. gambiae* as well [59, 60]. However, it important to note that generally there is local adaptation of malaria parasite to the vector which may influence the parasite’s reproductive rate.

### Anopheles and sero-epidemiology

A study by Londono-Renteria et al. determined the association between clinical malaria and mosquito bites measured by anti-*A. albimanus* antibodies in the Artibonite Valley [61]. The results of the study demonstrated that anti-salivary gland extract (SGE) immunoglobulin (Ig)G antibodies levels were higher in clinical malaria patients than uninfected people living in the same region. Furthermore, a significant positive correlation between the level of *anti*-*A. albimanus* IgG and IgM antibody levels was observed. Because IgM is indicative of recent exposure (in this case to mosquito bites) these findings suggest that antibodies against *A. albimanus* saliva, especially IgG, could potentially be useful markers of mosquito bite exposure in Haiti.

### Insecticides and resistance

Insecticides used or tested in Haiti for control of malaria vectors included the organochlorides: dieldrin [62] and DDT [63–65]; the organophosphates: dichlorvos [63, 65], malathion [66], and fenitrothion [67, 68]; the carbamate: Sevin [64]; and the pyrethroids: allethrin, phenothrin [68] and permethrin [69]. These insecticides have been used in various applications including indoor residual spray (IRS), ultra-low volume (ULV) spray and recently, permethrin in long-lasting insecticide-treated bednets (LLINs). In Haiti, the onset of resistance in *A. albimanus* was reported for dieldrin in 1960 [19, 62], DDT in 1968 [16], and fenitrothion in 1984 [67]. Based on historical testing, widespread resistance is thought to occur for DDT, while resistance to dieldrin [19] and fenitrothion [67] is considered to be low and perhaps focal. Recent, resistance testing of *A. albimanus* to permethrin in the Haitian Departments of Centre, Grand’Anse, and Sud, and resistance testing to various classes of insecticide in Sud-Est has not found any evidence of resistance using CDC bottle bioassays yet (unpublished data). Still, continued vigilance in required to anticipate development of resistance in response to insecticide pressure from chemical control efforts or agricultural insecticide usage.

### Vector control

Vector control in Haiti has a long history with various methods and outcomes.

#### Larval source management (LSM)

Larval source management refers to the targeted management of mosquito larval habitats, with the goal of suppressing mosquito larval and pupal abundance. Techniques used in LSM include environmental management and manipulation, larviciding, biological control or combinations of these methods [70]. A recent systematic review of malaria larval control in Africa, Asia, Europe showed that with the added benefit of targeting mosquito larvae, it was possible to reduce malaria cases by 75 percent in some sites [71].

In Haiti, all forms of LSM have been attempted, including, environmental management, gasoil mixed with 10 % volume gasoline, larvivorous fish (i.e. *Poecilia reticulata* and others), and chemical larvicides such as the organophosphate, temephos-50 % emulsion concentrate (Abate E-500). More recently the toxin from bacterium *Bacillus thuringiensis israelensis* (Bti) has been incorporated into larviciding activities. These have been implemented by SNEM and the PNCM/NMCP. These approaches have not been evaluated for parasitological or entomology impact [19], and none have been evaluated recently.

The earliest reports of organized vector control in Haiti, date back between 1919 and 1936 during the occupation of Haiti by the United States, where the US Navy Medical Services utilized traditional drainage, oiling and filling to protect deployed soldiers [16]. However, while drainage and ditching projects continued well into the 1940s, a drainage project in Petit-Goâve (the site of an epidemic focus) from 1969 to 1970 provides the most detailed information on larval control in Haiti [72, 73]. In response to an outbreak, which accounted for 25 % of all malaria cases nationwide from 1968 to 1970, a source reduction project, that included drainage, ditching, larviciding, and larvivorous fish was designed and implemented in 1969 for the area. When comparing the epidemic focus with the rest of the country before the outbreak (February 1968–April 1970) and after the outbreak (May 1970–July 1972), a 98 % reduction in slide positivity rates was achieved in the malaria focus area [73]. In 1972, Carmichael reported that the drainage project reduced the number of malaria cases in the area from 1500 cases to 9 cases, despite a 200 % increase in the whole country [43, 72]. It is important to note that the authors make no mention of enhancements in diagnosis and treatment access in the epidemic foci.

#### Indoor residual spraying (IRS)

IRS is the application of a long-lasting, residual insecticide to potential malaria vector resting surfaces such as internal walls, eaves, and ceilings of all houses or structures (including domestic animal shelters) where such malaria vectors might come into contact with the insecticide [74]. In Haiti, IRS has been evaluated for its ability to kill mosquitoes, mostly in experimental hut trials [46, 63, 65], partly due to the success of neighbouring Caribbean countries to achieve malaria elimination with this technique [75, 76]. Both widespread coverage (>80 % household coverage) and focal IRS has been attempted in Haiti [19, 75]. From 1962 to early 1963, it was thought that IRS contributed significantly to the reduction of malaria in Haiti with a 77 % reduction in slide positivity rate (SPR) (6.5 % in 1962 to 1.3 % in 1963); however, these efforts seemed to have been hampered by abnormally heavy regional rains from April to September and Hurricane Flora in October 1963 [75] causing the SPR to rise to 3.5 % in 1964. In 1966, Haiti managed to reduce the SPR to 0.1 % [2]. This was attributed to IRS with DDT in 760,000 homes, active case detection of approximately three million people, and providing chloroquine and other anti-malarials (i.e. pyrimethamine) to one million people [2, 75]. However, these gains were not sustained.

Feinstein reviewed Service National des Endémies Majeures (SNEM) entomological records from 1978 to 1988 for IRS activities [16]. Generally, his finding showed that there was widespread resistance to DDT in sites across the country but mosquitoes were susceptible to malathion and fenitrothion. IRS with fenitrothion was initially used in limited areas of Les Cayes in 1978 but was later scaled-up to include other areas. More resting mosquitoes were caught inside of unsprayed homes than sprayed homes. However, more mosquitoes were caught outside of sprayed homes than unsprayed homes though the total number of collected mosquitoes were similar. Mortality of caught mosquitoes held for 24 h was higher in sprayed homes than unsprayed homes. Human-bait catches were variable. The results in Feinstein report showed that sprayed homes had higher density of host-seeking/biting mosquitoes than unsprayed homes; this result is counterintuitive to what would have been expected. The report states that the mosquito density was steadily declining over the years in sprayed homes while unsprayed homes did not show this declining trend.

#### Space spraying

Space spraying refers to the outdoor release of insecticides through a fog or mist from ground or aerial applications [77]. Space spraying is the most compatible vector control approach for vectors that exhibit outdoor resting and biting behaviour [18]. There are three types of space spraying including: thermal fogging, ultra-low volume spraying and outdoor residual (barrier) spraying.

Truck-mounted (ULV) spraying with d-allethrin and d-phenothrin was piloted in Les Cayes, Haiti in 1987 to determine its impact on *A. albimanus* population density [68]. The ULV applications consisted of a 20 % emulsifiable concentrate of d-allethrin and d-phenothrin at a rate of 6:14 (w/w) (Pesguard 201, Sumitomo Chemical Co. Ltd.). Spraying occurred between 18:00 and 21:00 h at 1-week intervals by a Leco HD sprayer mounted on a pick-up truck. Caged adult *A. albimanus* were kept outside to determine 30-min knockdown rates and 24-h mortality rates. Two houses were selected, (one in the centre of the spray area and another at the border of the spray area) to determine *A. albimanus* population density. HLC was done indoors by two mosquito collectors from 19:00 to 19:45 and indoor resting collections were done by mouth aspiration for 15 min. One hundred percent knockdown and mortality was observed with caged mosquitoes. However, low numbers of mosquitoes were captured during indoor mosquito collections by mouth aspiration and HLC. The authors did observe an increase in mosquito numbers after they stopped spraying. They suggested ULV spraying may contribute to reduced mosquito population in Haiti.

A large-scale prospective study was designed to test the effects of aerial ultralow volume (ULV) application of malathion to interrupt *P. falciparum* transmission [66]. The study was conducted in 1972–1973, in the Miragoane Valley of Haiti. Malathion was sprayed at a dosage of 4.5 fluid ounces per acre in the morning. The spray reduced the populations of adult *A. albimanus* to less than 1 % of pre-spray levels and interrupted epidemic transmission of *P. falciparum* malaria [78]. Prior to spraying, the incidence of malaria was similar in sprayed and unsprayed areas (176.1 and 198.7 cases/month/10,000 population, respectively). After three months of weekly spraying, a ~71 % reduction in malaria incidence was observed between sprayed and unsprayed areas (16.8 cases/month/10,000 population in sprayed areas and 65.4 in unsprayed; p < 0.001) [79]. Mortality of certain groups of insects such as bees, flies, beetles, and butterflies was observed immediately following spray application; however, longer term impact of spraying was not assessed though it was thought that insect populations would rebound from neighbouring insects after spraying [80]. An evaluation on the impact on living fish, tree lizards, birds and bats concluded there was minimal impact [80] on these non-target vertebrate. Thermal fogging in Haiti started in the 1970s [19]; currently, vehicle-mounted sprayers and hand-held portable sprayers with malathion is in use. However, historical or contemporary entomological/parasitological evaluation of fogging efficacy under local conditions have not been done.

Barrier spraying/outdoor residual spraying was also assessed in the DR [53]. Barrier spraying is the application of high concentration insecticide applied directly to vegetation and other surfaces where a mosquito may land (e.g. under decks, gazebos, etc.). The pyrethroid, deltamethrin was sprayed aerially as a ultra-low volume application at a treatment rate of 17–19 g a.i./ha (active ingredient/hectare) in a radius of 500 m around two villages. The *A. albimanus* population was monitored by light-traps and human bait collections at both treated villages, compared with two similar untreated villages, up to nine nights post-treatment. The densities of female *A. albimanus* were reduced by ~95 % measured by UV light traps in the sprayed villages for at least eight nights. However, only 48 % reduction in mosquito density was seen when using HLCs. The authors do not state the reason for the difference between light traps and HLCs.

#### Bed nets

While the impact of bed nets has been extensively tested and proven in African transmission settings, few published studies have evaluated the impact of bed nets on malaria transmission reduction in areas where *A. albimanus* is the primary vector. Studies on insecticide-treated nets (ITNs) for the reduction and malaria transmission in Colombia, Ecuador, Guatemala, Nicaragua and Peru suggested that ITNs have a role in reducing malaria transmission [81–83].

In 2012, a national bed net distribution was done in response to reports of high rates of malaria transmission after the 2010 earthquake in Haiti. The Global Fund (GF) sponsored a national distribution of LLINs throughout the country (except Port-au-Prince) with the goal of two Olyset LLINs per household. The impact of this national distribution was assessed using a case–control study to determine if there as any association between bed net ownership and malaria illness confirmed by rapid diagnostic test (RDT) [69]. From September 2012–February 2014, 9318 patients, including 379 (4.1 %) RDT-positive patients, were enrolled across 17 health facilities in five departments in Haiti. Slightly more than half (57.1 %) of patients reported owning any bednet, with no difference among matched cases and controls. No difference was found in the proportion of cases and controls who reported using any bednet (34.5 vs 32.9 %, p = 0.56) or a campaign ITN (21.9 vs 19.5 %, p = 0.32) the previous night, or always using a campaign ITN in the two weeks before their illness (18.4 vs 18.5 %, p = 0.94). In a multivariate conditional logistic regression model, consistent use of a campaign ITN was not related to RDT positivity. Additional entomologic investigation found that *Anopheles* mosquitoes were 100 % susceptible to permethrin in the study areas, the insecticide used on campaign LLINs, and ITN retained knockdown and mortality efficacy for 12 and 20-month bed nets.

## Discussion

This review chronicles the vector research and control activities in Haiti for almost three quarters of a century starting from 1940 to 2015. A total of six references dealt with mosquito distribution, seven with larval mosquito ecology, 15 with adult mosquito ecology, three with ento-epidemiology, eight with insecticide resistance, one with sero-epidemiology and 16 with vector control. Much of the literature generated was during the time of eradication and shortly afterwards. There has been very little recent entomology research in Haiti with only four published studies [37, 41, 42, 61] and three scientific abstracts [35, 36, 69] available in the last 15 years. As Hispaniola moves towards malaria elimination by 2020, it will be necessary for Haiti to continuously update the knowledge-base of vector research and control by conducting routine entomological surveillance and evaluation, as much of the historical literature generated may not hold true as environmental landscapes continue to change.

Within the context of malaria elimination, the value of understanding the distribution and bionomics of *A. albimanus* in Haiti and its ability to sustain malaria transmission cannot be understated. While the literature on malaria vector behaviour in Haiti is somewhat limited and out of date, the studies and programmatic documents identified and contained herein provide a useful starting point for making rational vector control decisions. Below is a brief description of how this information could be used to inform decision making.

Haiti is an extremely heterogeneous and fragmented environment with a highly marked relief, despite being relatively small in geographical extent. The geographic landscape and topology differs from one micro-region to the next. Furthermore, annually rainfall distribution differs clearly from the northeast to the southwest. The human environment is also extremely varied, from the comfortable houses including the rural areas to very poor housing not only on the hills but also in the lowlands and towns. This heterogeneity complicates the control of malaria.

*Anopheles albimanus* is the primary malaria vector. In Haiti, while *A. albimanus* has been reported at high elevations (above 700 m a.s.l.) it is generally thought that *A. albimanus* and subsequently malaria transmission are rare in upland Haiti due to few suitable habitats for vectors [12]. When overlaying elevation data with available mosquito data, it is generally true that most studies were done around coastal areas, low-lying areas, and below 500 m a.s.l. for all species. A major question relates to whether transmission is occurring at elevations >500 m a.s.l., but is not being reported because of the remoteness and low population density in these high elevation areas.

The preferred biting location (indoors or outdoors) and peak biting times of malaria vectors is a fundamental area of research that needs to be updated immediately, as this will direct the type, timing, and scale of vector control that could be useful in an elimination strategy. While it is generally thought that *A. albimanus* has a tendency towards exophilly [43], results reported in this review demonstrated regional difference in behaviour ranging from high degree of exophilly in the north, to equal indoor and outdoor biting south (see Table 2). These results suggest entomological efforts to reduce transmission could benefit from different vector control approaches appropriate for the local situation. As host selection is influenced by host availability, it will also be important to collect information on human activity in the hours prior to bedtime to further understand how outdoor peak biting times interact with human host availability. However, indoor and outdoor biting in Haiti appears to peak in parallel, so indoor biting patterns in relation to human behaviour at those times could also provide useful information. Understanding which alternate host are available (e.g. cattle, pigs, goats and other ungulates) in areas of human settlement may also inform vector control approaches because these animals may divert mosquito bites from humans or increase the biting population of mosquitoes and increase the risk of being bitten.

A major challenge of vector control in Haiti is selecting approaches that will efficiently provide the maximum impact for the purpose of malaria elimination. IRS [75], ITNs [69], larval control, consisting of drainage and oil larviciding [72, 73] and aerial ULV spraying [79] are the only methods used in Haiti where disease reduction impact has been reported. Of the four methods, aerial ULV spraying is perhaps the most rigorous, using a controlled-before and after study design. The larval control study compared the surveillance data from an epidemiological focus with the rest of the country. The ITN study used a case–control design surveying fever cases from health facilities. IRS has never been formally evaluated in Haiti but surveillance data showed an ecological association with a reduction in transmission from the time IRS with DDT was implemented in 1962–1963, before the introduction of MDA in 1964.

IRS and ITNs are accepted as the standard in vector control and are used universally. Based on historical mosquito behaviour studies showing some indoor mosquito biting in Haiti, malaria transmission in Haiti likely occurs indoors to an extent. While IRS and ITNs may not interrupt transmission, they may still have a significant contributory role in reducing transmission. In Haiti, the impact of IRS to reduce malaria transmission has not been rigorously assessed, so it is difficult to unequivocally state the extent of impact. However, after a blood meal, indoor biting mosquitoes are likely to rest even briefly on walls where they can come into contact with insecticides. In northern Haiti, it was estimated that 12 % of indoor biting mosquitoes could be found resting the walls after a blood meal. In the context of malaria elimination, where malaria transmission must be driven down to zero, this 12 % may be significant to achieving that goal. However, it is important to note that acceptance and coverage of IRS achieved during earlier eradication campaigns may not be reproducible due to the difference in political will between now and the Duvalier regime (1957–1971). However, national coverage may not be required for elimination and judicious focal application of IRS may be achievable.

The public health benefits of ITNs have been exhaustively demonstrated and are acknowledged, mostly for Africa [84]. The one study conducted to test the effectiveness of LLIN-use to reduce malaria transmission in Haiti found no significant effect [69]. Other studies in the Americas have also suggested limited or equivocal effect of ITNs to reduce malaria transmission [85, 86]. While case–control studies are less ideal for evaluating the impact of ITN on malaria transmission compared to more robust analytical method, options for further ITNs evaluations in Haiti are limited by low transmission which adversely impacts time and cost needed to do more rigorous follow-up studies. Similar to IRS, to achieve malaria elimination in Haiti, judicious focal use of ITNs may be a better approach compared to any expanded distribution.

Larval control and aerial spraying are the other vector control approaches used in Haiti that have been evaluated for their ability to reduce malaria transmission. The project in Petit-Goâve suggested an added benefit of larval control to interrupting malaria transmission in Haiti. While larval control does not have the same constraints as IRS and ITNs, its impact is restricted to areas where larval mosquitoes mostly undergo development in sites that are few, fixed and findable [70]. For that reason, larval control is mostly seen as a supplementary intervention [70].

Haiti is one of the few places where aerial spraying was assessed to determine its potential in interrupting malaria transmission. Studies in Miragoâne, Haiti are perhaps the most rigorous of their kind suggesting a role in malaria elimination in areas where transmission may be particularly recalcitrant to conventional approaches. However, an area would have to be thoroughly assessed to determine the impact on apiculture (bee-keeping) and non-target pollinators. The financial and technical cost of larval control and aerial spraying are viewed as prohibitive to a country like Haiti and potentially carry adverse environmental impact that may be unpopular to the local population. However in order to drive malaria transmission down to zero these approaches should be judiciously considered and supported by donors to achieve the goal of malaria elimination.

The ongoing improvements to Haiti’s surveillance capacity, diagnostics and case management requires significant financial and technical investment as they serve as the pillars of malaria elimination in Haiti. In general, vector control interventions may have greatest impact if applied sensibly in response to surveillance data and in support of malaria case management. Therefore, there must be a balance in all these tools to achieve elimination.

Whether widespread or focal interventions are used, malaria vector populations will have to be monitored for insecticide resistance. Currently, insecticide resistance has not been detected. However, resistance testing is still limited; expansion of testing is ongoing in Haiti with more sites and more insecticides being tested.

As Haiti makes progress in eliminating malaria, it will be difficult to measure impact as fewer cases will be detected. For that reason, serological approaches that measure malaria exposure and mosquito biting may be a method to measure risk or intervention impact. For example, high antibody titer against salivary gland antigens for *A. albimanus* may suggest the population is at risk of malaria transmission and a vector control intervention may be required to mitigate that risk; the vector control interventions can also be assessed by looking at the antibody response to evaluate impact.

New vector control approaches [87] and re-emerging strategies [88, 89] are gaining more interest, therefore, consideration should be made for these interventions. Some activities that may have immediate impact in Haiti include: (1) attractive toxic sugar-baits which exploit the sugar-feeding behaviour of male and female mosquitoes [90–94]; (2) ivermectin, an endectocide, which is an antiparasitic drug that have been found to be active against both helminths, specifically filarial worms, and disease arthropods, and are safe for humans and animals [95–99]; (3) spatial repellency technologies, which refers to the use of airborne chemicals that induce a range of insect behaviours that results in a reduction in human–vector contact [100]; and (4) the re-emerging strategy of Integrated Vector Management (IVM) as a rational decision-making process to optimize the use of resources for vector control which importantly considers judicious application of larval control or other vector control combinations [88].

## Conclusions

This review provides information on vector-related aspects of malaria transmission and vector control in Haiti. The limited evidence-base on the effectiveness of different vector interventions suggests that heavy investment in any single vector control approach should be avoided. Rather, vector control interventions should be implemented strategically under well-defined scenarios and evaluated accordingly. Further, basic entomological monitoring provides the ground work necessary to optimize these vector control strategies. Therefore, the role of entomology in malaria elimination remains crucial for success in Haiti.

## Abbreviations

a.s.l.: above sea level
Bti: *Bacillus thuringiensis israelensis*
CDC: Centers for Disease Control and Prevention
CLT: CDC light trap
DR: Dominican Republic
EIR: entomological inoculation rate
ELISA: enzyme-linked immunosorbent assay
GF: Global Fund
GOH: Government of Haiti
HLC: human landing catches
Ig: immunoglobulin
IRS: indoor residual spraying
ITNs: insecticide-treated bed nets
IVM: integrated vector management
LF: lymphatic filariasis
LLIN: long-lasting insecticide treated bed nets
MDA: mass drug administration
NMCP: National Malaria Control Programme
PNCM: Programme national de Contrôle de la Malaria (Haiti)
RDTs: rapid diagnostic tests
SPR: slide positivity rate
SNEM: Service National d’Eradication de Malaria/Service National des Endémies Majeures (Haïti)
UVLT: ultra-violet light trap
ULV: ultra low volume spraying
UNICEF: The United Nations Children’s Fund
USAID: United States Agency for International Development
VC: vectorial capacity
WHO: World Health Organization
